# Vagus Nerve Schwannoma: A Case Report and Literature Review

**DOI:** 10.1002/ccr3.70469

**Published:** 2025-04-21

**Authors:** Kato Ronald, Kantu Ronald, Jacinta Ambaru, Dan Sekiwunga

**Affiliations:** ^1^ Gulu University Faculty of Medicine, Kampala Uganda Gulu Uganda; ^2^ Department of Internal Medicine, Faculty of Health Sciences Makerere University Kampala Uganda; ^3^ Department of Oncology Uganda Cancer Institute Kampala Uganda; ^4^ Department of Radiology, Faculty of Health Sciences Makerere University Kampala Uganda

**Keywords:** benign, bradycardia, schwannoma, tumor, Vargus nerve

## Abstract

Vagus schwannomas are rare, benign masses that arise in the cervical region but can develop anywhere along the Vagus nerve. In most cases, patients present in their third to sixth decades, with neck swelling and hoarseness; herein, we discuss a case report of a 41‐year‐old female who presented with slurred speech and locking of her tongue and was diagnosed with Vagus schwannoma. The patient had an inability to speak properly and locking of her tongue during speech, with pain to the right side of the neck as Intermittent, prompted by turning her head to the right and shooting pain to her shoulder, which is short‐lived, with a sensation of difficulty swallowing and talking that is alleviated by moving her head to a neutral position. On examination, there was mild neck stiffness, fasciculations of the tongue, and left deviation of the tongue with inability to swallow. Blood pressure was 128/70 mmHg with 42 bpm bradycardia, which was persistent on several readings, with a small right neck mass measuring 1 cm × 2 cm, non‐tender on palpation. Imaging studies showed a well‐defined ovoid lesion centered in the right retrostyloid parapharyngeal space measuring 3.3 × 2.1 × 3.8 cm with central T2 hyperintensities representing a nerve sheath tumor that appears stable in size and morphology. The histology report typically displayed a characteristic pattern with two distinct tissue types, Antoni A and Antoni B, with tightly packed spindle cells arranged in palisades around a central verocay body and a looser arrangement of myxoid matrix. Immunohistochemistry showed lesional cells had strong immunopositivity for S100 and were negative for CD34. Hence, the conclusion of a Vagus nerve schwannoma. Patient was sent to the radiation oncology team and was started on Cyberknife 25 Gy treatment in five fractions, which were completed and sent for re‐evaluation and follow‐up. There was no surgical intervention in this case due to the delicate anatomical location of the mass; hence, Cyberknife radiation was the best option for treatment. Vagus schwannomas are rare, benign masses that usually develop in the cervical region but can arise anywhere along the Vagus nerve. Patients may be asymptomatic, but in most cases, they present in their third to sixth decades; hence, they should be considered in patients with otherwise unexplained bradycardia, with a history of dysphagia, slurred speech, and cervical masses.

**Summary**

- Vagus nerve schwannomas are very rare benign tumors which present in third to fifth decade of life, and it is worth noting they must be highly suspected in patients presenting with dysphagia, slurred speech, and asymptomatic bradycardia with any cervical neck masses.
- Due to delicate anatomical locations, surgical excision remains a challenging mainstay of treatment in symptomatic patients.

## 1 Introduction

Most Vagus nerve schwannomas have been found to arise within the cervical course of the Vagus nerve. These tumors are usually asymptomatic; however, varied symptoms such as pain, dysphagia, and shortness of breath have been reported. Of note, there is a high reported incidence of peri‐perioperative vocal cord paralysis. Vagus schwannomas are rare, benign masses that usually develop in the cervical region but can arise anywhere along the Vagus nerve. Patients may be asymptomatic; however, in most cases, patients present in their third to sixth decades, with neck swelling, hoarseness, or cough.

## 2 Case Report

### 2.1 History and Physical Examination

41‐year‐old female who presented to us with inability to speak properly and locking of her tongue during speech with persistent headache. The patient described her pain to the right side of the neck as intermittent, prompted by turning her head to the right and alleviated by moving her head to a neutral position and shooting pain to the shoulder, back of head and sometimes pain is constant. Pain can be severe with difficulty turning her head and constant headache with burning sensation to the right side of the skull which is very short‐lived and sensation of difficulty swallowing and talking.

During the consultation, the patient started having a slurred speech with locking of her tongue. Later, she experienced blurred vision and difficult swallowing. There is no history of chronic illness like hypertension, diabetes, or malignancy. There are no episodes of seizures, syncope, dizziness, or upper extremity weakness, and there is no history of Ptosis or diplopia.

The patient was rushed to the emergency department and was started on some medications and investigations.

### 2.2 Intervention

#### 2.2.1 Physical Examination

The patient was in Fair general condition, not pale and not cyanosed. There were fasciculations of the tongue and left deviation of the tongue with inability to swallow. Laryngeal examination was unremarkable at this step. Blood pressure was 128/70 mmHg with 48 bpm bradycardia. The bradycardia was persistent on several readings. There was a mild right neck mass measuring 1 cm × 2 cm, non‐tender.

#### 2.2.2 Investigations

The patient was stabilized with Intravenous diazepam 2.5 mg and sent for Imaging studies.

**FIGURE 1:**
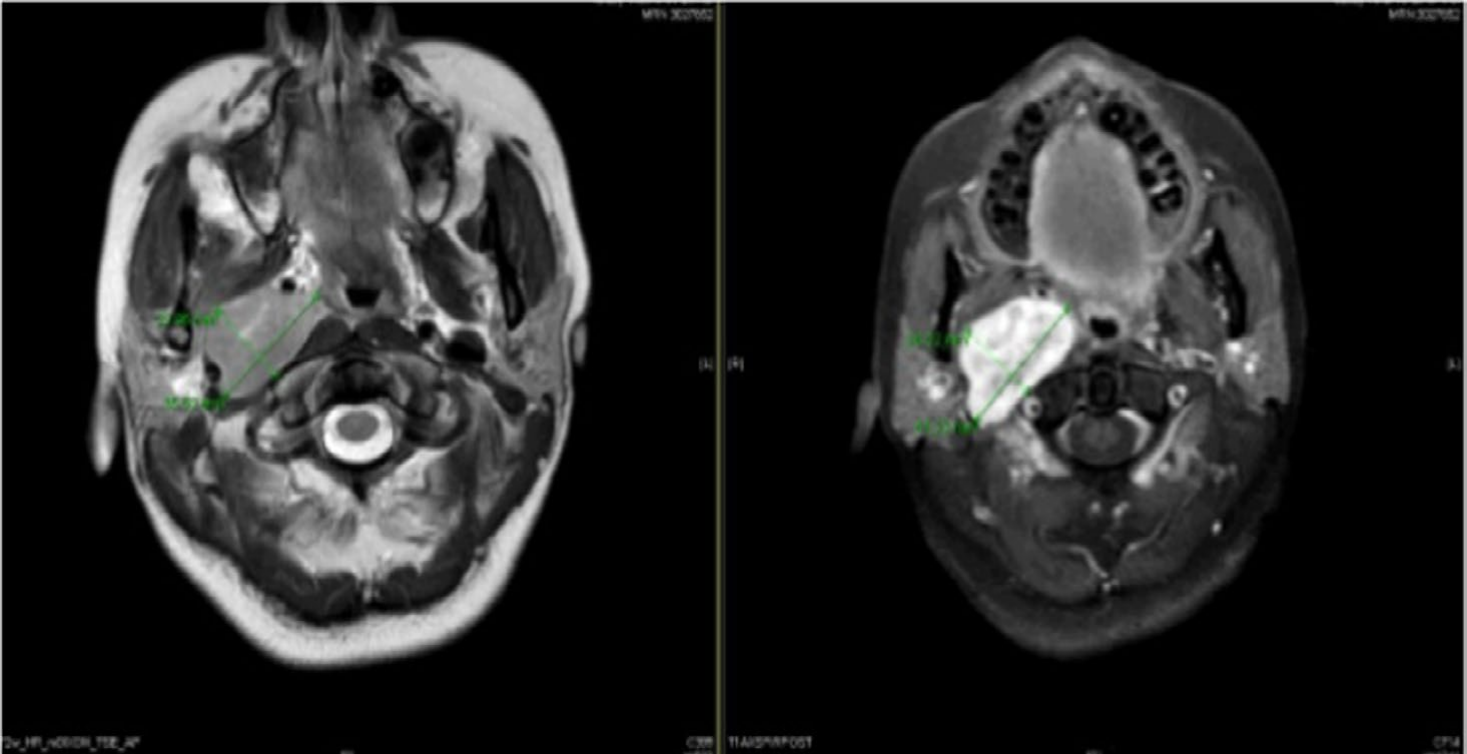
(A) Right without contrast and (B) left with contrast. Avidly enhancing well‐defined ovoid lesion centered in the right retrostyloid parapharyngeal space measuring 3.3 × 2.1 × 3.8 cm (AP × TV × CC) with central T2 hyperintensities representing a nerve sheath tumor that appears stable in size and morphology. This lesion results in splaying of the right ICA/ECA. Posterior displacement and flattening of the right internal jugular vein are noted. Mild mass effect on the right aspect of the oropharynx results in mild narrowing of the airway. Mass effect on the masticator space and parapharyngeal space occurs without evidence of invasion.

MRI scan with and without contrast was done showing an avidly enhancing well‐defined ovoid lesion centered in the right retro styloid parapharyngeal space with central T2 hyperintensities representing a nerve sheath tumor, that appears stable in size and morphology. This lesion results in splaying of the right ICA/ECA. Posterior displacement and flattening of the right internal jugular vein were noted with mild mass effect on the right aspect of the oropharynx that results in mild narrowing of the airway as shown in Figure 1A (right without contrast) and 1B (left with contrast), respectively.

**FIGURE 2:**
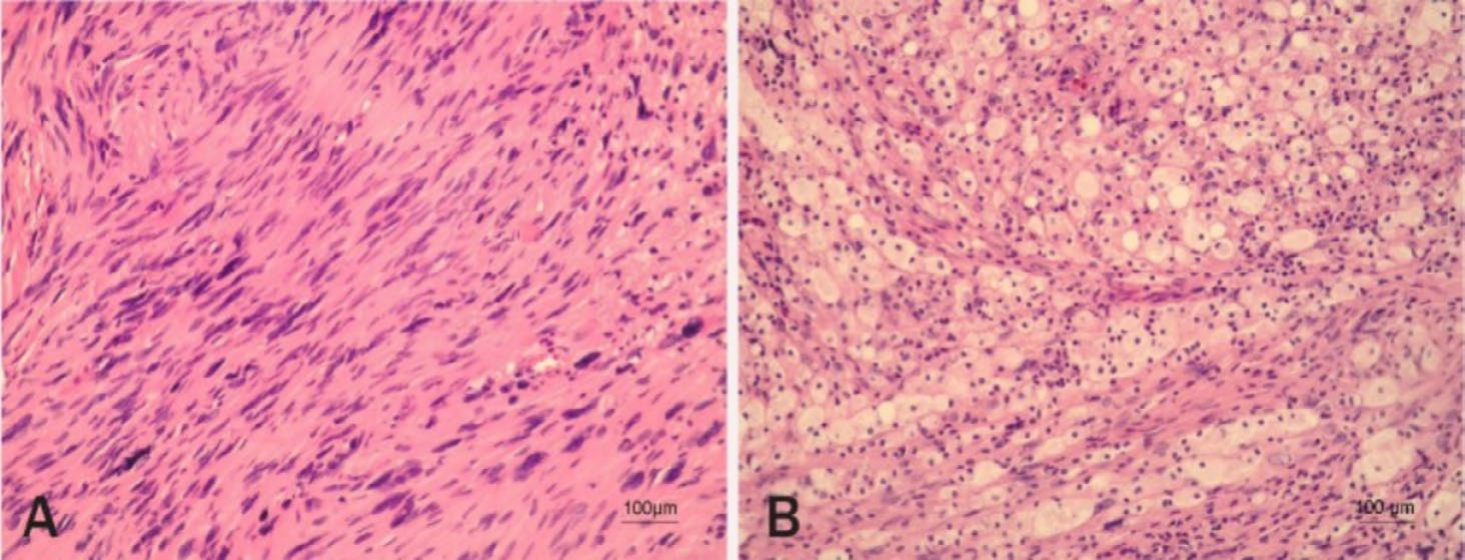
(A) Right and (B) left: Biopsy was performed and showed typically a characteristic pattern with two distinct tissue types, Antoni A and Antoni B, respectively. Antoni A areas showed tightly packed spindle cells arranged in palisades around a central verocay body with elongated nuclei displaced in concentric rows around eosinophilic material, while Antoni B areas exhibited a looser arrangement of cells within a myxoid matrix with detached fragments of skeletal muscle and fragments of fibroadipose and vascular tissue, with a conclusion of the impression of a nerve schwannoma.

Biopsy of the lesion was done and the histology report showed a characteristic pattern with two distinct tissue types: Antoni A and Antoni B, with tightly packed spindle cells arranged in palisades around a central verocay body and a looser arrangement of myxoid matrix, as shown in Figure 2A (Right) and 2B (Left), respectively.

**FIGURE 3:**
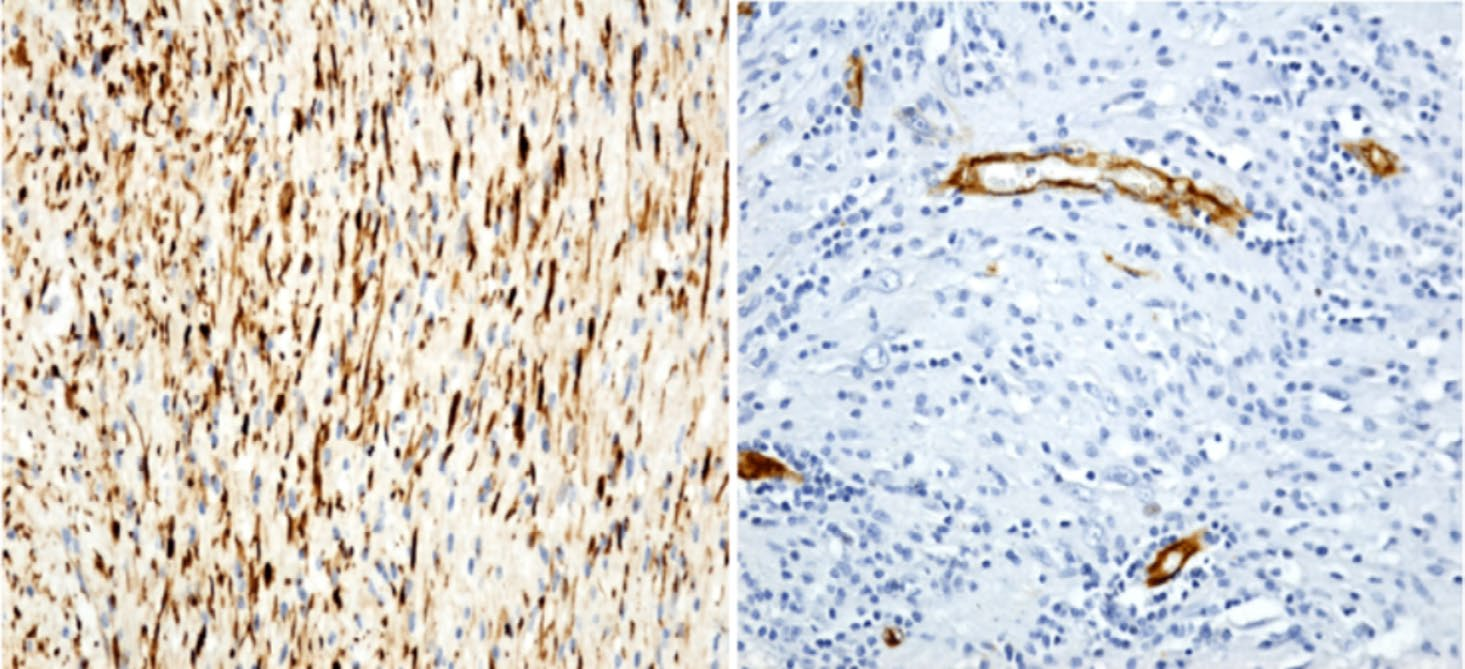
(A) (Right) showing positive for S100 and (B) (left) showing negative for CD34.

The Immunohistochemistry performed shows that the lesional cells which showed a strong immunopositivity for S100 and were negative for CD34. EMA is negative. Ki‐67 proliferative index is 1% as shown in Figure 3A (right) and 3B (left).

Due to the delicate anatomical location of the tumor, surgical resection was not possible as it would pose a significant mortality risk to the patient; thus, the patient was sent to a radiation oncologist. The Radiation team started her on Cyberknife 25 Gy treatment in five fractions, which were completed, and the patient sent for re‐evaluation.

### 2.3 Post‐Cyberknife Treatment With Surveillance Scan

**FIGURE 4:**
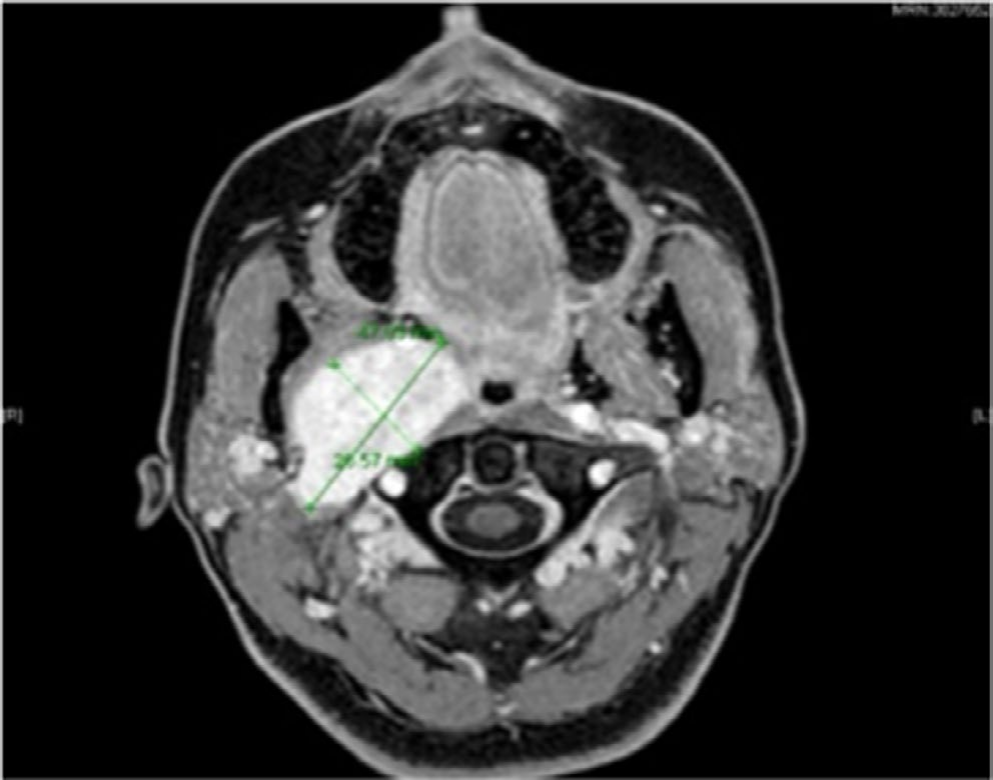
Showed a redemonstration of a T2 hyperintense, lobulated, well‐defined lesion within the right retro‐styloid parapharyngeal space measuring 4.2 × 2.5 × 4.5 cm but that has increased in comparison to the prior MRI. When it measured approximately 3.4 × 1.9 × 3.7 cm and measured similarly.

MRI Imaging performed at 1.5 Tesla. ProHance 16 mL Intravenous Showed a redemonstration of a T2 hyperintense, lobulated, well‐defined lesion within the right retro‐styloid parapharyngeal space measuring 4.2 × 2.5 × 4.5 cm but that has increased in comparison to the prior MRI, when it measured approximately 3.4 × 1.9 × 3.7 cm, which measured similarly as shown in Figure 4.

**FIGURE 5:**
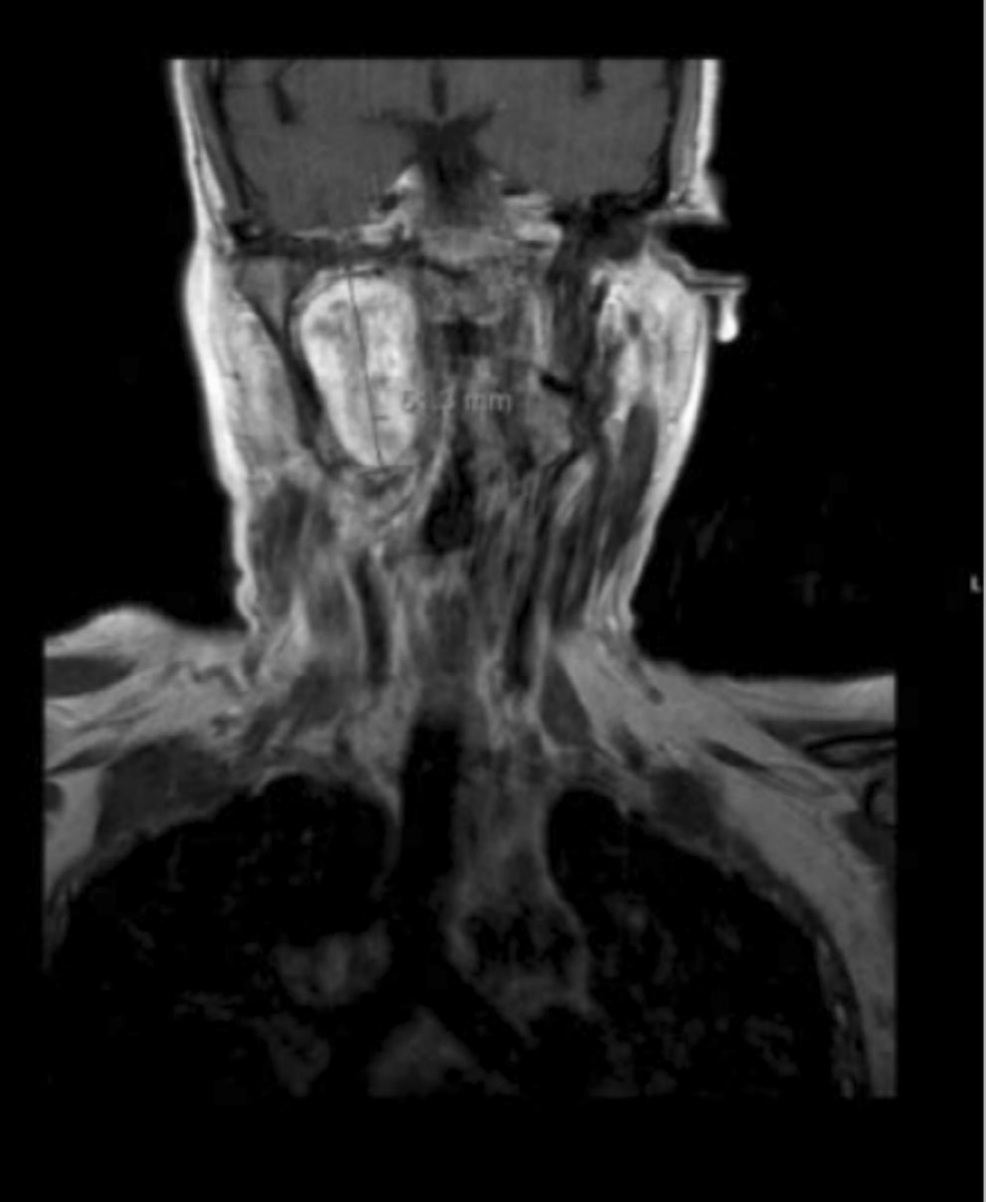
Showing a similar mass effect on internal and external carotid arteries, right‐sided mass effect on the oropharynx, and narrowing of the right internal jugular vein that is posterolaterally displaced by the mass and avidly enhancing well‐defined lobulated T2 hyperintensity in the right aspect of the lateral tongue representing a schwannoma with mass effect on the regional vessels without evidence of thrombus.

There is similar mass effect due to such as splaying of the internal and external carotid arteries, right‐sided mass effect on the oropharynx, and narrowing of the right internal jugular vein that is posterolaterally displaced by the mass. There is mass effect on the right pterygoid muscles and narrowing of the retromolar trigone fat as shown in Figure 5.

### 2.4 Differential Diagnoses

(1) Carotid body tumor.

#### 2.4.1 Role of Radiological Parameters and Role of Radiotherapy and Out‐Come for Extracranial Schwannomas

Radiological imaging plays a critical role in the diagnosis, characterization, and surgical planning of extracranial vagus schwannomas. Key imaging modalities and parameters include:

1. CT‐Scan

Appearance: Well‐defined, homogeneous, oval or fusiform masses along the course of the vagus nerve.

Enhancement: Moderate to strong enhancement with contrast.

Bony changes: May show smooth erosion or remodeling of adjacent bony structures, such as the jugular foramen or carotid sheath.

2 MRI

T1‐Weighted Images: Hypointense to isointense compared to muscle.

T2‐Weighted Images: Hyperintense due to high water content in the tumor.

Contrast‐Enhanced T1: Heterogeneous or homogeneous enhancement, often with a “target sign” (central hypointensity with peripheral hyperintensity).

Relation to Surrounding Structures: MRI is superior in delineating the tumor's relationship to adjacent vessels, nerves, and soft tissues.

3 Ultrasound

Appearance: Well‐circumscribed, hypoechoic masses with posterior acoustic enhancement.

Doppler: May show vascularity within the tumor.

4 Angiography

Rarely used but may show displacement of adjacent vessels without significant tumor vascularity.

### 2.5 Role of Radiotherapy

Radiotherapy is not the primary treatment for vagus schwannomas but may be considered in specific scenarios:

Inoperable tumors: For patients with high surgical risk due to medical comorbidities or tumor location.

Recurrent tumors: When complete surgical resection is not achievable.

Residual disease: Adjuvant radiotherapy may be used to control residual tumor growth.

Stereotactic radiosurgery (SRS): Techniques like Gamma Knife or CyberKnife are preferred for small, well‐defined tumors, offering precise targeting with minimal damage to surrounding tissues.

### 2.6 Outcomes of Radiotherapy

#### 2.6.1 Tumor Control

Radiotherapy, particularly SRS, has shown high rates of local tumor control (80%–95%) with minimal progression over long‐term follow‐up. Growth arrest or shrinkage of the tumor is commonly observed.

#### 2.6.2 Functional Preservation

Radiotherapy is associated with a lower risk of cranial nerve deficits compared to surgery, making it a favorable option for tumors in critical locations.

### 2.7 Complications

Rare but may include radiation‐induced neuropathy, fibrosis, or secondary malignancies.

The risk of complications is higher with conventional radiotherapy compared to SRS.

### 2.8 Long‐Term Outcomes

Patients often maintain good quality of life with preserved vagal function.

Regular follow‐up with imaging is essential to monitor tumor response and detect recurrence.

### 2.9 Conclusion and Prognosis

Vagus schwannomas are rare, benign tumors that typically arise along the course of the vagus nerve, most commonly in the cervical region. Although some patients may remain asymptomatic, many present in their third to sixth decades with symptoms such as dysphagia, slurred speech, and cervical neck masses. Unexplained bradycardia in conjunction with these symptoms should raise clinical suspicion for vagus schwannomas, making it an important differential diagnosis. Early recognition and appropriate diagnostic imaging are crucial for timely management and to prevent potential complications. Surgical excision remains the mainstay of treatment, with favorable outcomes when performed by experienced surgeons. A multidisciplinary approach is often necessary to optimize patient care and ensure long‐term follow‐up.

## 3 Review of Literature

Vagal schwannomas are considered a true challenge for clinicians. It is difficult to obtain a specific preoperative diagnosis because the signs and symptoms are commonly not specific. In the early stage of the disease, the most common symptom is a solitary, slow‐growing mass in the neck that can be palpated along the medial border of the sternocleidomastoid muscle. There are also cases of schwannomas found incidentally in asymptomatic patients [1].

The term “schwannoma,” first introduced in 1935 by Stout, identifies a benign tumor with sporadic malignant degeneration arising from cranial, peripheral and autonomic nerve sheath cells. It represents 5% of all benign soft tissue tumors, equally affects both genders and typically shows higher incidence between the third and fifth decade. Head and neck localization is infrequent, and vagal origin in that region is unusual [2, 3, 4].

Schwannomas and neurofibromas are often misdiagnosed as both being benign peripheral nerve sheath tumors. The main difference is that schwannomas are eccentric to the nerve of origin, whereas neurofibromas are spindle‐shaped and located in the center of the perineurium [5].

Schwannomas are tumors that are of Schwann cell origin, of central, peripheral, or autonomic nervous system. Schwannomas do not arise from the optic and olfactory nerves as they lack those cells. Perineural fibroblast tumor, nerve sheath tumors, and neurilemmomas are its other nomenclatures. Around 25%–45% of these tumors are present in the head and neck region. Schwannoma presents in the 4th to 6th decades of life with no sex predilection.

A slow‐growing swelling and lesion without pain and neurological features are the common presenting symptoms in schwannoma. In the parapharyngeal space, the vagus nerve is the most common nerve of origin.

There are only a few studies describing extracranial, non‐vestibular, head and neck schwannomas, and the majority are individual case reports [6].

Twenty‐five cases were included in the study, among whom 13 were females and 12 were males (1:1 ratio). The mean age was 46 years (range 22–74 years). Except for two patients who suffered from neurofibromatosis, all the others had a solitary head and neck schwannoma [7].

Unilateral neck swelling was seen in 19 (76%) patients. Of the nineteen tumors located in the neck region, 10 cases (53%) had a left‐sided mass, and 9 cases (47%) had a right‐sided mass. Nasal mass (8%), as well as ear mass (4%), were left‐sided, whereas cheek swelling (4%) presented on the right side. All the 25 patients underwent preoperative radiological evaluation [7].

Around 25%–45% of these tumors are present in the head and neck region. In the head and neck region, the most common occurrence is in the parapharyngeal space. Only around 54% of head and neck schwannomas arise from the sinonasal tract [8].

Most of the literature states that schwannomas are prevalent between the third and fifth decades of life. However, a study done by Shrikrishna et al. found more prevalence in males (3:1) with the highest incidence in the second decade of life [9].

A retrospective review of radiographic cross‐sectional images of parapharyngeal schwannoma found that schwannomas arising from cervical sympathetic chains displace both carotid and jugular vessels without separating them. The study also revealed that the carotid and internal jugular vessels were separated in the case of vagal schwannoma [10].

Shoss et al. have recommended high‐resolution CT to identify the size and extent of the tumor. It also helps demonstrate the degree of vascularity of the tumor and differentiate between malignant and benign conditions [11].

A study done in Japan on functional nerve preservation in schwannomas found that motor schwannoma enucleation with intraoperative EMG guidance had no complications post‐surgery, whereas in sympathetic schwannomas, chances of postoperative neurological dysfunction like Horner syndrome were seen. Intracapsular enucleation preserved nerve function by more than 30%, as compared with complete tumor resection. A study had stated that the postoperative nerve palsy rates were 100% with resection and division of the nerve of origin, 67% with intracapsular enucleation, and 50% with debulk operations [12].

A 10‐year retrospective study on 46 patients conducted in Shanghai, China, by Wang et al. revealed that all the tumors examined were benign. Females were seen to be more affected than males. 52% of the cases presented as an asymptomatic mass with a mean size of 4.5 cm. The brachial plexus was the most common nerve of origin in the study [13].

In a retrospective study by Gavin et al., the mean age at diagnosis was 48 years with a female predominance. 76% presented with complaints of unilateral neck swelling. Vagus and cervical sympathetic chains were the most common nerves of origin in this study. No recurrence was noted in 2 years of follow‐up [14].

A retrospective study by Bondi et al. which included 18 patients revealed that painless neck mass was the most common presenting complaint in patients with head and neck schwannoma. Female predominance was noted with a mean age of 42.1 years. Half of the group underwent ultrasonography with fine‐needle aspiration, which was diagnostic in 30% of the cases. MRI was diagnostic in 77% of the cases [15].

Some authors propose as a strategy for functional preservation in the treatment of extracranial schwannomas of the head and neck: the use of an electromyography (EMG) system during tumor resection could help to prevent motor nerve paralysis [12].

According to Smith et al., vagal paragangliomas were the biggest tumor in their study cohort with a size of 5.3 cm (±1.9 cm). They further mentioned that the most common mode of treatment is surgical excision with preoperative embolization. The resultant complications include complete unilateral vagal nerve paralysis together with additional glossopharyngeal, hypoglossal nerve, and cervical sympathetic chain disturbances [16].

Heyes et al. suggested that surgical excision is the standard treatment of choice for most vagal schwannomas, but they stressed that nowadays, the contemporary management evolves toward a conservative modality due to the high morbidity of the surgery [17].

## Author Contributions

**Kato Ronald:** conceptualization, formal analysis, investigation, methodology, project administration, resources, supervision, validation, writing – original draft, writing – review and editing. **Kantu Ronald:** project administration, resources, writing – review and editing. **Dan Sekiwunga:** investigation, methodology. **Jacinta Ambaru:** resources, writing – review and editing.

## Consent

An informed consent was obtained from the patient's mother to publish this report in accordance with the Journal's patient consent policy. Written Informed consent “I have been explained to the importance of this article and how it's publication will benefit future doctors and I totally consent to its publication.”

## Conflicts of Interest

The authors declare no conflicts of interest.

## Data Availability

The information in this manuscript can be found on the website below https://pubmed.ncbi.nlm.nih.gov.
